# Access to Community Living Infrastructure and Its Impact on the Establishment of Community-Based Day Care Centres for Seniors in Rural China

**DOI:** 10.3390/ijerph15061184

**Published:** 2018-06-06

**Authors:** Man Li, Renyao Zhong, Shanwen Zhu, Lauren C. Ramsay, Fen Li, Peter C. Coyte

**Affiliations:** 1 School of Public Administration, East China Normal University, 3663 Zhongshan Road, Shanghai 200062, China; 52164403003@stu.ecnu.edu.cn or lemon.li@mail.utoronto.ca (M.L.); ryzhong297@hotmail.com (R.Z.); poqianliuwu@sina.com (F.L.); 2 Institute of Health Policy, Management and Evaluation, University of Toronto, Health Science Building, 155 College Street, Suite 425, Toronto, ON M5T 3M6, Canada; lauren.ramsay@theta.utoronto.ca; 3 Toronto General Hospital Research Institute, University Health Network, 200 Elizabeth Street, Toronto, ON M5G 2C4, Canada; 4 Canadian Centre for Health Economics, 155 College Street, Toronto, ON M5T 3M6, Canada

**Keywords:** Chinese rural community, infrastructure access, day care centres for seniors

## Abstract

Community-based day care centres play an important role in service delivery for Chinese seniors. Little research has examined how community living infrastructure has influenced the establishment of these day care centres in rural communities. The purposes of this study were: (1) explore regional differences in community living infrastructure; and (2) to examine the impact of such infrastructure on the establishment of day care centres for Chinese seniors in rural communities. The data were derived from “The Fourth Sample Survey on the Living Conditions of Elderly People in Urban and Rural China (2015)”. The establishment of at least one day care centre was the outcome of interest, which was dichotomized at the community level into the establishment of at least one day care centre or the absence of any day care centres. Logistic regression analysis was employed to examine the impact of various community living infrastructural characteristics on the establishment of day care centres. The results showed that of the 4522 rural communities surveyed in 2015, only 10.1% had established at least one day care centre. Community living infrastructural characteristics that were significantly associated with the establishment of day care centres were the availability of cement/asphalt roads, natural gas, tap drinking water, sewage systems, and centralized garbage disposal. Our findings suggest that the significant association between community-level characteristics, especially community living infrastructure, and the establishment of rural day care centre for seniors may inform policy decision making.

## 1. Introduction

In China, as a country profoundly influenced by Confucianism, care for the elderly is primarily dependent on the tradition of filial piety [1]. Over the few decades, however, with the dramatic aging of its population, and additionally, the one-child policy, fertility decline, and changes in family structure and social attitudes have weakened the family support role [2,3]. Encouraged by the potential job opportunities and economic expectations, an increasing number of young labours have moved away from rural villages to urban centres, but this migration has left their elderly parents at home alone [4,5]. The elderly is often referred to in the literature as “empty nest” elders. Their care needs and quality of life have received significant attention and discussion in recent years [6,7,8]. As a consequence, the care needs of seniors and the increased demand for social support for families has increased simultaneously.

In China, community-based day care centres for seniors has been recognized as the main formal care institution to provide support to families. These centres provide daily services, such as medical and rehabilitation services along with assistance with activities of daily living for seniors who live in local communities. According to the “Chinese 12th Five-Year Plan of Social Services for Seniors” promulgated by the Chinese state council in 2011, seniors day care centres have been seen to play a fundamental role in promoting access to equality services for seniors in all communities [9]. By the end of 2016, there were over 35,000 day care centres established in China, an increase of 34.6% compared with 2015 [10]. Nonetheless, there has been uneven development of day care centres between urban and rural areas, as well as among rural regions. A range of studies have assessed the determinants of such inequities in the development of day care centres from an individual perspective [11,12,13,14,15]. For example, some studies have examined the association between personal characteristics (such as sociodemographic factors, social economic status, presence of caregivers, insured type and health conditions) and the development of day care centres [11,12,16,17]. However, a limited set of empirical research has focused on the role of community-level characteristics and their impact on inequities in the development of day care centres.

Fundamentally, access to services not only depends on who people are (the individual characteristics) but also where they live (the community characteristics) [18]. As a middle-income country, China’s aging population has also resulted in a greater emphasis on services accessibility, which is of great significance for less developed areas. Since community-based day care centres have been seen as the main formal care institution to improve the accessibility of care for elders, the “establishment or existence” of a day care centre is the prerequisite for realizing its function. It is our contention, that despite significant focus on market-based reforms in China, inequities in access to basic infrastructure between rural and urban areas have influenced various aspects of rural development, including of the development of community-based services for seniors. According to The Economic Development Institute (EDI) of the World Bank, in developing countries, the basic infrastructural services are services that allow citizens to participate in normal social activities. Such services include housing, transportation, water, sanitation, solid waste disposal and energy for cooking and lighting [19]. In China, rural infrastructure may be divided into four categories, including agricultural production infrastructure (such as infrastructure supporting modern agricultural production and irrigation), rural community living infrastructure (such as safe drinking water, natural gas, rural roads, local public transport and electricity), rural social development infrastructure (such as educational institutions, health care centres, and social interaction facilities), and ecological and environmental infrastructure (such as the infrastructure used in protecting natural and ecological resources) [16,20]. The development of each of these four rural types of infrastructure has varied across communities and may be one of the reasons why access to elder care services has been uneven across rural regions [16].

As the main services provided by day care centres are mainly related to elderly daily care [21], we are interested in the role of community-level living infrastructure and their impact on the establishment of adult day centres for seniors. This study will analyze the relationship between access to community living infrastructure and the establishment of day care centres for seniors in rural Chinese communities. The two main objectives of this study are to: (1) explore regional differences in community living infrastructure; and (2) to examine the impact of such infrastructure on the establishment of day care centres for Chinese seniors in rural communities. 

## 2. Materials and Methods

### 2.1. Data

The data were derived from “The Fourth Sample Survey on the Living Conditions of Elderly People in Urban and Rural China (2015)”. This survey, which was initiated in 2000, was conducted by China’s National Working Commission on Aging. In 2015, the survey was the first to cover all 31 of China’s mainland provinces. In this survey, 466 cities/counties, 1864 towns/districts, and 7456 communities were randomly selected from 31 provinces. The survey was divided into two parts: a community questionnaire (in which the response rate of 99.4% resulted in 7408 responses from 7456 possible responses) and an individual questionnaire for the elderly aged 60 and above (in which the response rate of 98.4% resulted in 22,017 responses from 22,368 possible responses). In this study, our unit of analysis was the community, thus we used data that were collected through the community questionnaire. The community-level survey was based on a combination of structured and semi-structured questions. These questions asked about social-geographic characteristics (such as community location, basic infrastructure (such as road types), services for seniors (such as health care), support for the provision of community aging services (such as funding), and institutions that provided services for seniors (such as day care centres). The respondent to the community questionnaire was the leader of the bureaucracy responsible the provision of community aging services, which resulted in high response rate and more reliable data than that derived from the individual-level survey. Among the 7408 effective community responses, there were 2886 (38.9%) urban communities and 4522 (61.1%) rural communities. 

### 2.2. Measures

#### 2.2.1. Outcome Measure: Establishment of a Day Care Centre for Seniors

In this study, the unit of analysis was at the community level. The community was defined as an administrative region that is recognized to have a diverse population living and sharing common infrastructure, facilities, and services. The establishment of at least one day care centre in the community was the outcome of interest, and it was dichotomized into a binary outcome: the establishment of at least one day care centre was assigned a value of one and the absence of any day care centres was assigned a value of zero. Readers should note that, in our study, “establishment” means “existence”. We want to emphasize the established (existence) or non-established (non-existed) status of a day care centre. Such status was measured through responses to the question “Did this community have a day care centre for local seniors?” with a response yes or no.

#### 2.2.2. Explanatory Variables: Rural Community Living Infrastructure

In this study, our research question concerned how community living infrastructural characteristics influenced the establishment of day care centres for seniors. In China, according to the “Chinese 12th Five-Year Plan of Social Services for Seniors” plan, the establishment of a day care centre for seniors in each community is the prerequisite for delivering health care to local elderly residents. In our study, the conceptual model for the selection of potential predictor variables that may be associated with the establishment of day care centres was based on The Model of Shortages of Sufficient Health Care in Rural Areas” developed by Weinhold and Gurtner [22]. Six sets of factors that included both individual and community characteristics were identified in this model, including the physical/infrastructural, professional, educational, social–cultural, economic and political reasons [22]. In our study, we have focused on physical/infrastructural. The reasons for the emergence of provider shortages have primarily been infrastructural deficiencies, such as those related to inadequate transport, communication infrastructure and a lack of social and cultural facilities. The choice of appropriate measured variables of rural community living infrastructure also referenced the EDI report about the basic infrastructure services in developing countries [19] and the reality of the types of Chinese rural infrastructure [16]. Moreover, the data availability was considered in the choice of those variables.

Therefore, the selection of the main explanatory variables and potential covariates were the following. First, the main set of explanatory variables were the physical/infrastructural dimension related to community living infrastructural characteristics, such as road type, cooking fuel, drinking water type, sewage system, and garbage disposal. Road type was divided into five categories: cement road, asphalt road, dirt road, gravel road, and others; cooking fuel was separated into five categories: natural gas, coal, electricity, firewood, and others; drinking water was divided into four categories: well water, tap-water, surface water, and others; sewage system was a binary “yes” or “no” variable; garbage disposal was classified into three categories: centralized processing, self-processing, and others. Second, referencing the other five sets in “The Model of Shortages of Sufficient Health Care in Rural Areas”, our selected community-related covariates were: the availability of a community activity space for seniors, the presence of a community medical care centre, community acreage, proportion of seniors, proportion of seniors with low-socioeconomic status, proportion of seniors without children and in-come, proportion of seniors living alone, funding for seniors services, barrier-free facilities and community location. There were some missing values for some of the selected explanatory variables and covariates, but the proportion of missing values were extremely low; less than 5% of the sample. To deal with these missing values, we followed the methods used in Guerriere et al. [23] and Yang et al. [24] wherein observations with missing data were recognized as a single separate category. In our study, continuous variables were reclassified as categorical variables by dividing the data into quartiles, as performed by Cai et al. [25]. 

### 2.3. Statistical Analysis

The potential predictors associated with the establishment of day care centres for seniors were analyzed. First, Pearson’s Chi-square tests were used to examine whether differences in the establishment of day care centres and each potential independent variable was statistically significant [26]. Second, univariate logistic regression was applied to explore the simple relationship between community living infrastructure and establishment of a day care centre. Third, multivariate logistic regression analysis was employed to analyze the association between basic infrastructure and the establishment of a day care centre after controlling for various covariates. Fourth, Hosmer-Lemeshow was used to test the goodness of fit of the model [26]. Fifth, we used the variance inflation factor to assess collinearity. In this study, the selection of statistical methods was based on our conceptual model and research objectives. All of the results from the univariate and multivariate models were presented as odds ratios (ORs), and 95% confidence intervals (CIs). An OR above one indicates that the specific predictor was more likely to be associated with the establishment of day care centres for seniors in a rural community. The statistical analyses were conducted using SPSS 23.0 for Windows (IBM, Armonk, NY, United States).

## 3. Results

Table 1 reports the descriptive statistics for the study variables and the distribution of the categorical variables stratified by the presence or absence of a senior day care centre. The majority of the communities had asphalt and cement roads (88.4%). Most of the communities used natural gas (33.3%) and firewood (38.7%) as their main cooking fuels. Over 50% of the communities were equipped with tap water, but only a small proportion of communities had a sewage system (19.6%). Most communities (61.4%) processed garbage centrally. About 86.2% of communities provided a community activity space for seniors. Most of the communities did not have a local medical care centre (89.8%). Only a small proportion of communities were located at the centre of town (9.5%), and 43.3% of communities were near a town. The remaining 47.2% of communities were located far from a town. Only a small proportion of communities were equipped with barrier-free facilities, such as a barrier-free elevator (0.6%) and barrier-free toilets (7.6%). Many communities reported that they had no funding dedicated to senior services (62.2%). In terms of population characteristics, the difference in the distribution of the population of seniors among communities was minor, but more than half of communities had a high proportion of seniors who were living alone (56.5%). Based on Pearson’s Chi-square tests, whether a community had at least one day care centre or not was significantly associated with all of the infrastructural characteristics listed. Of the 4522 communities in this study, only 10.1% of communities had a day care centre for seniors. Those communities with a day care centre had better access to community infrastructure than those without a day care centre.

Table 2 shows the univariate ORs for the likelihood that communities had a day care centre for seniors. Since our focus was on the assessment of the impact of community living infrastructure on whether communities had a day care centre, we only present results based on community infra-structural characteristics. All of the community living infrastructural variables were significantly associated with the presence of at least one day care centre. 

Day care centres were more likely to be established in communities with asphalt and cement roads; tap water as the main source of drinking water; natural gas or electricity as the main cooking fuels; those with centralized garbage disposal; and those with a sewage system.

Table 3 shows the statistical results of the multivariate logistic regression analysis, which analyzed the association between potential predictor variables and the establishment of a day care centre for seniors. In our study, the main explanatory variables had a significant association with the establishment of a day care centre.

Multivariate logistic regression results show that those communities equipped with asphalt or cement roads, tap water, natural gas or electricity, centralized garbage disposal and sewage system are more likely to establish a day care centre for seniors. For example, for communities with sewage system (OR: 1.9, 95% CI: 1.4 to 2.5) was more likely to have a day care centre for seniors. However, communities with firewood (OR: 0.4, 95% CI: 0.3 to 0.7) as the main cooking fuel was significantly less likely to have established a day care centre for seniors. Furthermore, when examining the role of community-related covariates, the multivariate logistic regression analysis demonstrated that community acreage, proportion of seniors living alone, and proportion of seniors with low-socioeconomic status had no statistically significant association with the establishment of day care centre for seniors. However, communities equipped with wheelchair access (OR: 1.7, 95% CI: 1.2 to 2.4) and communities with more supportive funding (OR: 3.8, 95% CI: 2.7 to 5.5) more likely to establish day care centres. Further, although the community location, the proportion of seniors in the population, the proportion of seniors who have no children, and those without income had a statistically significant association with the establishment of day care centre for seniors, the association was weaker.

## 4. Discussion

Ensuring equitable access to elderly care in all communities is an enduring concern for public policy-makers [27]. In China, community infrastructural characteristics have influenced various aspects of rural development. Inequalities in community-level health care access may exist for a variety of reasons, such as economic and social-cultural factors [28,29,30]. To our knowledge, our study is the first to examine the association between community living infrastructural characteristics in rural Chinese communities and the establishment of day care centres for seniors. The purpose of our research was to assess the association between a range of variables connected with community living infrastructural characteristics and the establishment of day care centres. 

We found that, based on the dataset used for this empirical study, only 10.1% of 4522 rural communities had day care centres for seniors in 2015. Subsequently, our logistic regression analysis results demonstrated that the establishment of these centres was significantly related to community living infrastructural characteristics including the type of road, cooking fuel used, the source for drinking water, the presence of a sewage system, and the occurrence of a garbage disposal system. This result is similar to, but not exactly the same as, those of some related empirical studies in developed countries. In the United States, significant differences in access to health care also exists between rural and urban regions, similarly, insufficient public transportation was recognized as one of the barriers to accessing health care in the rural regions of the US, while other community living infrastructural variables were not found to be associated with access to such care. What is interesting, the poor availability of community broadband internet services was shown to be related to the accessibility of US rural health care [31]. However, in current rural China, the internet accessibility was not recognized as a community living infrastructure. Similar findings have been reported in a Canadian study, in which telephone access was associated with the rural-urban differences in healthcare-seeking behaviours [27]. Two possible explanations may help understand these differences between China and Western developed countries. On one hand, the pace of urbanization may be associated with important differences among communities among both Western countries and in China [32]. In Western countries, the path to urbanization was completed decades ago, however, in China, urbanization levels are just 57.4% in 2016 [10]. One of the major differences in the level of urbanization has been in the degree of imbalance in infrastructural development. For example, by the end of 2016 in urban and rural regions, the availability of natural gas varied from 95.75% to 22.0%, respectively; similarly, the equivalent relative availability of other infrastructural resources was 98.4% and 68.7% for tap water, and 93.4% and 20.0% for sewage systems [28]. In rural Chinese areas, most communities had a low level of urbanization, and the limited use of gas, tap water, and sewage systems, which has as associated the establishment and management of day care centres. On the other hand, on average, likely internet access in rural China is limited; it is more akin to high-level service rather than as a form of basic community living infrastructure. Furthermore, compared with Western countries, the development of modern rural communities in China has been a very recent phenomenon. Originally, in China, the main functions of rural communities were governance and administration, without any reference to social services including services for seniors [33].

We also found other potential predictors of the establishment of day care centres for seniors. Specifically, such centres were associated with specific funding support for senior services [34], the proportion of seniors in the population [35], and access to barrier-free facilities [36]. These findings were consistent with other Western studies. However, in our review of the literature we found a paucity of research that has examined these specific predictors, such as the availability of a community activity space for seniors and the presence of a community medical care centre, which are not included in any of six factors in our conceptual model. This void in the literature may because of the quantum difference in the development status of China compared to Western countries. In Western countries, the development of community-based services has moved beyond infrastructural needs to the provision of services such as long-term care [37], end-of-life care [38] and palliative care [39]. 

There are several limitations associated with our study. First, due to the lack of relevant data, we were unable to control for other potential variables that may have associated with the establishment of day care centres. For example, we did not examine the impact of the availability of human resources on the establishment of such centres. Some studies have reported that a short-age of professional caregivers and nurses, possibly due to lower levels of remuneration in rural communities, retard the establishment of day care centres [17]. Second, readers should note that our study focused on how community-living infrastructural characteristics impact the establishment of day care centres, not how such characteristics influence the services used. Services utilization at day care centres may be influenced by the type of services provided [14,40], service quality [13], and other factors. Third, because we used cross-sectional data from 2015, we were unable to make any longitudinal comparisons regarding the establishment of day care centres. Since the publication of China’s 12th five-year seniors’ services development plan, day care centres have been recognized as formal caregiving services for seniors and are now supported by public policy. Consequently, data on day care centres was collected by “The Fourth Sample Survey on the Living Conditions of Elderly People in Urban and Rural China” in 2015 for the first time. The use of this data in our study has the potential to influence government policy regarding the establishment of day care centres for seniors in rural Chinese communities.

## 5. Conclusions

In our study, of that only 10.1% of rural communities had established at least one day care centre for seniors, despite growth in the need for senior services to support the dramatically aging population of contemporary China. We found that access to community living infrastructure, which included cement/asphalt roads, natural gas, tap drinking water, sewage systems, and centralized garbage disposal, were significantly associated with the establishment of day care centres for seniors. Our findings suggest that access to community living infrastructural resources should be recognized as a significant factor when examining the potential predictors of the establishment of day care centre for seniors in rural China. Furthermore, our findings may contribute to expanding our applied conceptual model when it is used to describe access to health care in developing countries. According to our study, a range of community living infrastructural factors, as well as other important variables (such as the availability of a community activity space for seniors and the presence of a community medical care centre) may need to be taken into account when examining the relationship between community-level features and access to health care. Our findings suggest that the significant association between community-level characteristics, especially the community living infrastructure, and the establishment of rural day care centre for seniors may inform policy decision making. 

## Figures and Tables

**Table 1:** Descriptive statistics of the total study communities.

Variables	% of All Communities (*n *= 4522)	Day Care Centre		*p*-Value		
		NO (*n* = 4067)	YES (*n* = 455)		X^2^	*df*
Road type				0.00	35.62	5
asphalt road	12.4%	10.3%	30.5%			
cement road	76.0%	76.9%	68.2%			
dirt road	5.5%	6.1%	0.2%			
gravel road	4.7%	5.2%	1.1%			
others	1.2%	1.3%				
missing	0.2%	0.2%				
Cooking fuel				0.00	128.70	6
natural gas	33.3%	29.4%	68.4%			
coal	11.5%	11.9%	8.1%			
electricity	15.4%	16.0%	10.5%			
firewood	38.7%	41.7%	11.8%			
others	0.7%	0.7%	0.4%			
missing	0.4%	0.4%	0.7%			
Drinking water				0.00	96.98	4
tap water (pipe)	57.7%	54.3%	88.6%			
well water	33.9%	36.6%	9.4%			
surface water	6.4%	7.0%	1.5%			
others	0.9%	1.0%	0.2%			
missing	1.0%	1.1%	0.2%			
Centralized-heating system				0.01	9.09	2
no	98.1%	98.6%	93.2%			
yes	1.3%	0.7%	6.4%			
missing	0.7%	0.7%	0.4%			
Sewage system				0.00	220.30	2
no	79.1%	83.3%	41.0%			
yes	19.6%	15.3%	58.6%			
missing	1.3%	1.4%	0.4%			
Garbage disposal				0.00	131.86	3
Centralized processing	61.4%	58.0%	92.1%			
self-processing	37.3%	40.7%	7.5%			
others	0.7%	0.8%	0.2%			
missing	0.6%	0.6%	0.2%			
Available activity space for seniors				0.00	37.31	2
no	9.3%	10.1%	1.7%			
yes	86.2%	85.2%	95.7%			
missing	4.5%	4.7%	2.6%			
Medical care institution around community				0.00	27.93	1
no	89.8%	91.1%	78.7%			
yes	10.2%	8.9%	21.3%			
Community acreage				0.00	24.11	4
quartile1	9.4%	8.8%	14.4%			
quartile2	24.3%	24.0%	27.2%			
quartile3	31.4%	30.9%	35.6%			
quartile4	34.9%	36.3%	22.8%			
Seniors proportion				0.00	212.72	4
quartile1	21.6%	22.8%	11.1%			
quartile2	28.7%	30.0%	17.0%			
quartile3	26.9%	27.4%	22.3%			
quartile4	22.8%	19.8%	49.6%			
Proportion of low-socioeconomic status				0.00	190.60	4
quartile1	11.0%	8.6%	32.2%			
quartile2	24.7%	23.5%	35.1%			
quartile3	31.3%	32.7%	19.1%			
quartile4	32.9%	35.1%	13.5%			
Proportion of seniors without child and income				0.00	128.29	4
quartile1	11.1%	10.0%	21.3%			
quartile2	19.6%	17.7%	36.9%			
quartile3	33.2%	33.9%	27.3%			
quartile4	36.1%	38.5%	14.4%			
Proportion of seniors living alone				0.00	58.14	4
quartile1	17.6%	16.3%	31.0%			
quartile2	25.9%	25.9%	26.6%			
quartile3	27.9%	28.5%	21.2%			
quartile4	28.6%	29.3%	21.2%			
GDP of the cities/counties *				0.00	245.37	3
quartile1	27.1%	28.0%	19.1%			
quartile2	29.6%	31.7%	10.3%			
quartile3	25.5%	26.0%	21.1%			
quartile4	17.8%	14.3%	49.5%			
Working funding for seniors services				0.00	383.66	2
0	62.2%	66.4%	24.5%			
1–10,000	22.4%	21.8%	28.1%			
10,000+	15.4%	11.8%	47.4%			
Wheelchair access				0.00	160.32	1
no	88.3%	90.7%	66.9%			
yes	11.7%	9.3%	33.1%			
Barrier-free elevator				0.00	21.45	1
no	99.4%	99.6%	97.7%			
yes	0.6%	0.4%	2.3%			
Barrier-free toilet				0.00	116.84	1
no	92.4%	94.0%	77.3%			
yes	7.6%	6.0%	22.7%			
Community location				0.00	40.02	2
centre of town	9.5%	8.6%	17.3%			
near the town	43.3%	43.1%	45.3%			
far from town	47.2%	48.3%	37.4%			

* GDP was measured by the cities/counties level where communities belongs.

**Table 2:** Univariate odds ratios for likelihood of community with at least one day care centre.

Variables	Vs.	*p*-Value	OR	LCL	UCL
Road type		0.000			
cement road	asphalt road	0.000	0.410	0.275	0.612
dirt road		0.043	0.120	0.015	0.935
gravel road		0.003	0.135	0.036	0.504
others		0.998	0.000	0.000	
missing		0.999	0.000	0.000	
Cooking fuel		0.004			
coal	natural gas	0.396	1.246	0.750	2.068
electricity		0.061	0.648	0.411	1.021
firewood		0.001	0.471	0.305	0.727
others		0.926	1.086	0.187	6.307
missing		0.503	0.466	0.050	4.355
Drinking water		0.000			
well water	tap water	0.000	0.340	0.220	0.525
surface water		0.010	0.205	0.062	0.683
others		0.998	0.000	0.000	
missing		0.998	0.000	0.000	
Sewage system		0.006			
yes	no	0.004	1.659	1.181	2.330
missing		0.275	0.290	0.031	2.682
Garbage disposal		0.006			
Self-processing	Centralized processing	0.001	0.425	0.261	0.692
others		0.539	1.931	0.236	15.777
missing		0.999	0.000	0.000	

OR: Odds Ratio; LCL: Lower 95% confidence limit; UCL: Upper 95% confidence limit.

**Table 3:** Multivariate odds ratios for likelihood of community with at least one day care center.

Variables		Vs.	*p*-Value	OR	LCL	UCL
Road type			0.000			
cement road		asphalt road	0.000	0.399	0.280	0.569
dirt road			0.020	0.091	0.012	0.689
gravel road			0.003	0.185	0.060	0.570
others			0.998	0.000	0.000	
missing			0.999	0.000	0.000	
Cooking fuel			0.000			
coal		natural gas	0.265	1.300	0.820	2.061
electricity			0.054	0.671	0.447	1.007
firewood			0.000	0.447	0.302	0.662
others			0.983	0.981	0.173	5.570
missing			0.436	0.421	0.048	3.722
Drinking water			0.000			
well water		tap water	0.000	0.363	0.245	0.539
surface water			0.018	0.315	0.121	0.821
others			0.998	0.000	0.000	
missing			0.998	0.000	0.000	
Sewage system			0.000			
yes		no	0.000	1.855	1.368	2.516
missing			0.306	0.340	0.043	2.677
Garbage disposal			0.002			
self-processing		centralized processing	0.000	0.426	0.274	0.661
others			0.821	1.269	0.161	10.017
missing			0.998	0.000	0.000	
Available activity space for seniors			0.016			
yes		no	0.006	7.267	1.754	30.111
missing			0.070	4.702	0.883	25.031
Medical care institution around community						
yes		no	0.970	1.008	0.669	1.518
Community acreage			0.403			
quartile2		quartile1	0.232	1.383	0.812	2.354
quartile3			0.114	1.514	0.905	2.533
quartile4			0.109	1.553	0.907	2.659
Seniors proportion			0.001			
quartile2		quartile1	0.770	1.075	0.663	1.742
quartile3			0.195	1.369	0.851	2.202
quartile4			0.002	2.120	1.314	3.419
Proportion of low-socioeconomic status			0.403			
quartile2		quartile1	0.232	1.383	0.812	2.354
quartile3			0.114	1.514	0.905	2.533
quartile4			0.109	1.553	0.907	2.659
Proportion of seniors without child and income			0.028			
quartile2		quartile1	0.752	0.939	0.634	1.390
quartile3			0.012	0.560	0.356	0.881
quartile4			0.247	0.746	0.454	1.225
Proportion of seniors living alone			0.535			
quartile2		quartile1	0.544	0.875	0.569	1.346
quartile3			0.563	0.879	0.568	1.360
quartile4			0.163	0.700	0.424	1.155
Supportive funding for seniors services			0.000			
1–10,000		0	0.000	2.384	1.690	3.364
10,000+			0.000	3.830	2.665	5.502
Barrier-free facilities						
Wheelchair access	yes	no	0.003	1.685	1.197	2.373
Barrier-free elevator	yes	no	0.339	0.467	0.098	2.223
Barrier-free toilet	yes	no	0.649	1.096	0.740	1.622
Location			0.040			
near the town		centre town	0.020	0.597	0.387	0.922
far from town			0.247	0.761	0.480	1.208

OR: Odds Ratio; LCL: Lower 95% confidence limit; UCL: Upper 95% confidence limit.
